# Spinal curvatures of yoga practitioners compared to control participants—a cross-sectional study

**DOI:** 10.7717/peerj.12185

**Published:** 2021-09-16

**Authors:** Małgorzata Grabara

**Affiliations:** Institute of Sport Science, Jerzy Kukuczka Academy of Physical Education, Katowice, Polska

**Keywords:** Hatha yoga, Thoracic kyphosis, Lumbar lordosis, Plurimeter-V

## Abstract

**Purpose:**

The angles of thoracic kyphosis and lumbar lordosis determine the spinal alignment in the sagittal plane. The aim of this study was to compare the thoracic kyphosis and lumbar lordosis of male and female yoga practitioners with non-practicing participants and to determine the possible dependencies between sagittal spinal curvatures and somatic parameters, time spent on yoga exercise, and undertaking other physical activities in yoga practitioners.

**Methods:**

The study involved 576 women and 91 men ages 18–68 years (mean = 38.5 ± 9) who were practicing yoga, and 402 women and 176 men ages 18–30 years (mean = 20.2 ± 1.3) as a control group. The angles of thoracic kyphosis and lumbar lordosis were measured using a Plurimeter-V gravity inclinometer.

**Results:**

The two-way ANOVA demonstrated the influence of group (*p* < .0001) and sex (*p* = .03) on the angle of thoracic kyphosis, as well as the influence of group (*p* < .0001) and sex (*p* < .0001) on the angle of lumbar lordosis. It was noted that yoga practitioners had less pronounced thoracic kyphosis and lumbar lordosis and were more often characterized by normal or smaller thoracic kyphosis and lumbar lordosis than students from the control group. In yoga practitioners, the angle of thoracic kyphosis was positively correlated with age, body mass, BMI, and undertaking other forms of physical activity. The angle of lumbar lordosis was negatively correlated with body height and body mass.

**Conclusions:**

The results suggest that yoga exercises can affect the shape of the anterior-posterior curves of the spine and may be an efficient training method for shaping proper posture in adults.

## Introduction

Human posture changes during the course of one’s life and reflects one’s physical and mental health. Posture depends on a variety of factors, *i.e.,* age, gender, lifestyle, occupation, muscle balance, kinesthetic sense, somatic parameters, as well as genetic and environmental factors (Araujo et al., 2014; DrzaL-Grabiec & Snela, 2012; Grabara & Pstragowska, 2008; Lang-Tapia et al., 2011; Zwierzchowska, Tuz & Grabara, 2020). Proper posture should be characterized by symmetry in the frontal and transverse planes, and the spinous process line should overlap with the mechanical axis of the spine (Grabara, 2020).

In the sagittal plane, the spine has four physiological curves, *i.e.,* lordotic in the cervical and lumbar regions and kyphotic in the thoracic and sacral regions. The determination of standards for posture in the sagittal plane is difficult and largely depends on the measurement technique used. However, assessing spinal posture is highly important. The alteration of anteroposterior curvatures could result the dynamic imbalance of the spine and distinction of muscle tone and may lead to lower back pain (LBP) (Glassman et al., 2005; Mac-Thiong et al., 2011). Hyperkyphosis is one of the most prevalent spinal disorders, and an increase in thoracic kyphosis can be associated with higher spinal loads and trunk muscle force in the upright position. Hyperkyphosis has been linked to the impairment of the respiratory function, decrease in physical function, cervical pain, subacromial pain syndrome, and headaches (Shamsi et al., 2014). Hyperkyphosis has also been associated with low values of hamstring flexibility and a lack of abdominal and paravertebral strengthening (Czaprowski et al., 2018; Gonzalez-Galvez, Gea-Garcia & Marcos-Pardo, 2019). The flattening of the lumbar lordosis increases the strain on passive elements of the spine (De Carvalho et al., 2010). Decreased lumbar lordosis may be associated with disc degeneration at the L_5_/S_1_ intervertebral disc level. Previous studies revealed that intradiscal pressure was inversely proportionate to the degree of lumbar lordosis (De Carvalho et al., 2010; Adams & Hutton, 1985). Increased lumbar lordosis may be responsible for the high incidence of osteoarthritis in the hip and knee joints (Adams & Hutton, 1985). Hyperlordosis is also associated with a shortening of the psoas iliac and the lack of abdominal and paravertebral strength (Czaprowski et al., 2018; Gonzalez-Galvez, Gea-Garcia & Marcos-Pardo, 2019).

Regularly undertaking physical activity (PA) can affect posture, including sagittal spinal curvatures. Previous studies assessing posture in athletes have indicated specific spinal adaptations in several types of athletes such as volleyball players (Grabara, 2020; Grabara, 2015), handball players (Grabara, 2014a), basketball players (Grabara, 2016), cyclists (Muyor, Lopez-Minarro & Alacid, 2011), skiers (Alricsson & Werner, 2006), rhythmic gymnasts (Kums et al., 2007), dancers (Kruusamae et al., 2015), hockey (De Baranda et al., 2020) and wrestlers (Rajabi et al., 2008). Specific exercise programs or other forms of PA such as Nordic Walking (Hanuszkiewicz, Wozniewski & Malicka, 2021), Pilates (González-Gálvez et al., 2020) a hatha yoga can also have an influence on posture.

Hatha yoga (physical yoga exercise) includes specific exercises (yoga poses) called “asanas.” An adequately adopted asana is a stable pose with an optimal (correct), *i.e.,* axial and symmetric, alignment of body parts in order to avoid overloading the passive elements of the locomotor system. Concentrating on the correct alignment of body parts during yoga exercise leads to the shaping of proper posture. Studies focusing on the effects of yoga exercise on posture have suggested that it leads to improvements in posture (Grabara & Szopa, 2011b; Grabara & Szopa, 2011a; Grabara, 2014b; Greendale et al., 2009). These improvements particularly relate to the reduction of excessive thoracic kyphosis in the elderly (Grabara, 2014b; Greendale et al., 2009) and in young adults (Grabara & Szopa, 2011a), as well as decreased excessive lumbar lordosis in young women (Grabara & Szopa, 2011a).

The current study comprised a large population of people who regularly practiced yoga and a population of young, healthy people in the posture stabilization period as a control group. To the best of the author’s knowledge, such studies have not been previously conducted.

The study aimed to accomplish the following: (1) to compare sagittal spinal curvatures between persons practicing yoga exercises and non-practicing participants, and (2) to determine the possible dependencies between spinal posture and somatic parameters, time spent on yoga exercise, and undertaking other physical activities in yoga practitioners.

## Materials & Methods

### Participants

The study involved 576 women and 91 men who were practicing yoga (yoga group) ages 18–68 years (mean = 38.5 ± 9), and 402 women and 176 men ages 18–30 years (mean = 20.2 ± 1.3) as a control group (posture stabilization period).

The age range in the yoga group (YG) was as follows: 41% women and 45% of men were 18–35 years old; 38% of women and 28% of men were 36–45 years old; 18% of women and men were 46–55 years old; 3% of women and 9% of men were over 55 years old. The declared training experience in YG varied between 1–15 years and was as follows: 14% of participants had 1–2 years of yoga training, 42% had 2–5 years, 29% had 5–10 years, and 15% had over 10 years of yoga training. The declared volume of yoga training peer week varied between 60–840 min (mean = 269 ± 127.5). The mean frequency of yoga training was three times per week (min. 1, max. 7), and the mean volume of one yoga session was 80 min (min. 20, max. 180). A few of the observed yoga practitioners (20%) declared that they undertook other forms of physical activity at least twice a week, *i.e.,* walking, swimming, jogging, cycling, aerobics, Pilates, or muscle-strengthening exercises.

The study inclusion criteria for YG were the following: status as a student of postgraduate studies in “relaxation and yoga” or a student of a yoga instructor’s course, minimum one year of yoga practice, undertaking regular yoga practice (minimum 60 min per week for the last year), consent to participate in the study. The study exclusion criteria for YG was undertaking another form of PA in a greater weekly duration than yoga training.

The control group (CG) was comprised of university students who were neither undertaking yoga nor professional sports training. The study inclusion criteria for CG were the following: status as a student, non-athlete, no sports history, consent to participate in the study.

The sample size included all possible persons for testing, taking into account the experimental criteria.

### Methods and procedures

This study was approved by the local bioethics committee (no. 3/2012) and conformed to the standards established by the Declaration of Helsinki. All participants were informed about the type and aim of the study and they gave verbal consent. The study was conducted from March 2013 to February 2020.

Sagittal spinal curvatures were measured using a Plurimeter-V gravity inclinometer (Dr. Rippstein, Zurich, Switzerland). It is a precision-built, liquid pendulum inclinometer with a dial that can be fully rotated 360 degrees. That noninvasive technique proved to be highly reliable and valid in the assessment of spinal curvatures (Grabara, 2016; Grabara & Szopa, 2015). The correlation of the spinal curvature measurements performed with the Ripstein plurimeter *versus* digital photography was *r* = 0.949, p < .0001 for thoracic kyphosis and *r* = 0.951, *p* < .0001 for lumbar lordosis (Stolinski et al., 2014).

The participants were asked to adopt a habitual posture without any postural correction as previously described in Grabara (2016). Specifically, participants were instructed to stand in a straight position on the floor with their eyes and ears in line horizontally, arms relaxed at the side of the body, and feet shoulder-width apart. The plurimeter was set in the “zero” position at the seventh cervical vertebra (C_7_) and the upper part of the thoracic spine. The thoracic kyphosis angle (ThKA) was taken at the level of the thoracolumbar junction. The plurimeter was then reset at this height and again placed at the thoracolumbar junction (Grabara, 2016). Following this, the lumbar lordosis angle (LLA) was measured. The value 30 ± 5 was considered as normal kyphosis and lordosis (Zwierzchowska, Tuz & Grabara, 2020; Grabara, 2016). Measurements were carried out by one examiner with a precision of 1 degree. Measurements of the ThKA and LLA were performed twice, and when a substantial discrepancy occurred (over 5°), the angles were measured for a third time; in the first case, the second measurement was taken for analysis. In the other case, the extreme measurements were rejected (Grabara, 2016).

Basic anthropometric parameters (body height and body mass) were also measured and BMI was calculated. Body height (BH) was measured to the nearest 0.5 cm using medical scales with a mechanical height rod. Body weight (BW) was measured using the Tanita-410 Body Composition Analyzer (with a precision of 0.1 kg).

Yoga practitioners also completed a questionnaire to provide information on their yoga training experience, volume and frequency of yoga sessions, and undertaking of other forms of physical activity.

### Statistical analysis

Results are expressed as the mean, median, standard deviation, and minimum and maximum values of the parameters. The somatic parameters between persons practicing yoga exercises and non-training participants were compared using the t test. Sagittal spinal curvatures were compared using the two-way analysis of variance (ANOVA). A post-hoc Tukey’s test for different n was performed when a significant main effect was detected. For comparing the frequency of the occurrence of proper spinal curvatures, Pearson’s chi^2^ test of independence was applied. To determine the potential relation of the studied parameters, Pearson’s r correlation coefficient (linear correlation coefficient), two-way ANOVA, and linear multiple regression analysis were applied. The level of significance was set at p ≤ .05. Statistical analysis was undertaken using Statistica ver. 13, TIBCO Software Inc.

## Results

The basic somatic parameters of the studied participants are presented in Table 1. Male yoga practitioners showed values that were significantly lower than the control participants, whereas female yoga practitioners had a significantly lower BMI than the control participants (Table 1).

The means, standard deviations, median, and minimum and maximum values of the spinal curvatures angles are presented in Table 2.

The two-way ANOVA demonstrated the influence of group (*p* < .0001; effect size = .02; power analysis = .998) and sex (*p* = .03; effect size < .01; power analysis = .583) on the angle of thoracic kyphosis, as well as the influence of group (*p* < .0001; effect size = .04, power analysis = 1) and sex (*p* < .0001; effect size = .11; power analysis = 1) on the angle of lumbar lordosis. The test did not reveal a significant group *vs.* sex interaction in both thoracic kyphosis and lumbar lordosis angles. The results of the post hoc analysis are presented in Tables 3–4.

**Figure 1:**
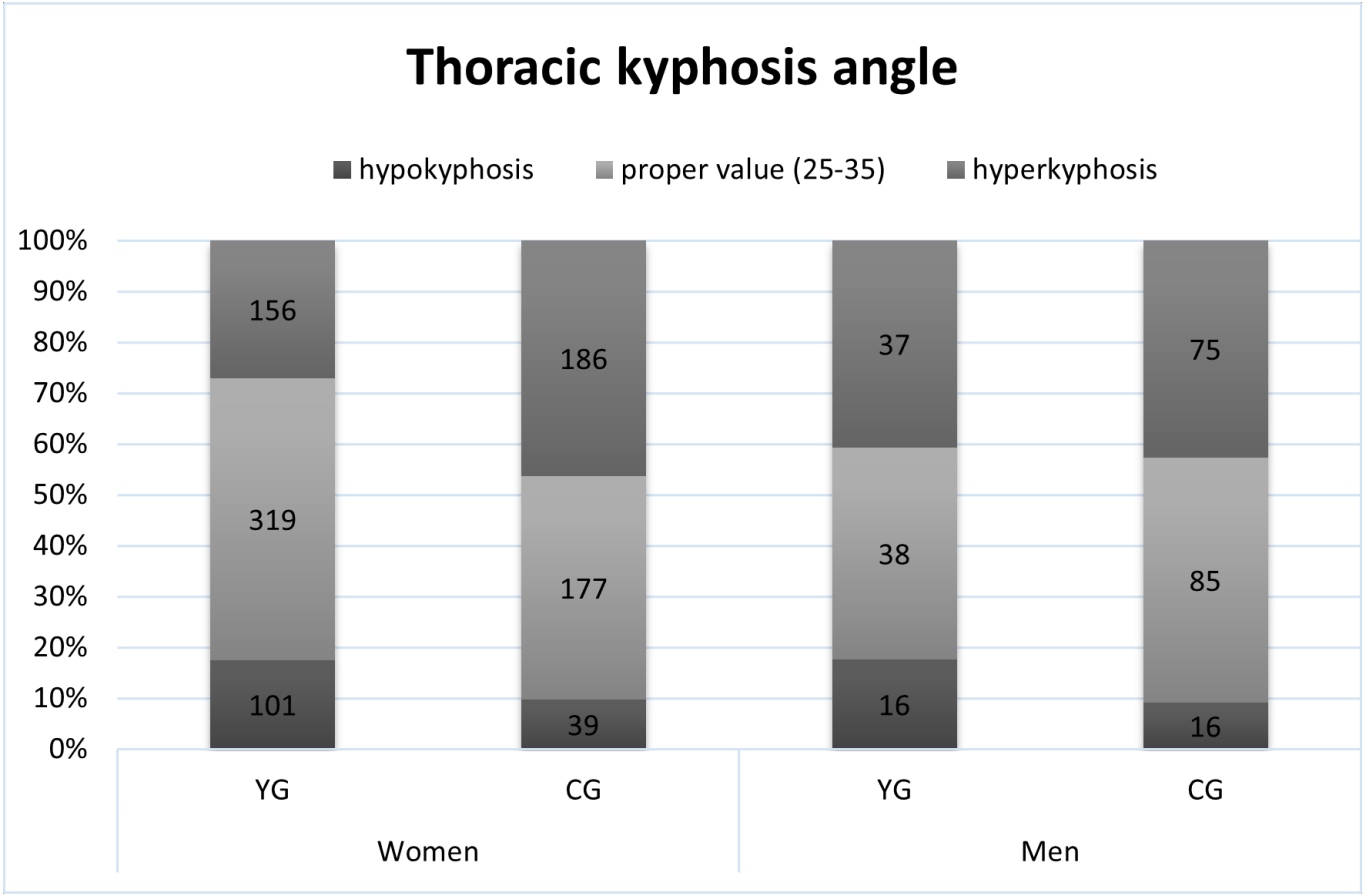
The occurrence of proper thoracic kyphosis, hypokyphosis and hyperkyphosis.

**Figure 2:**
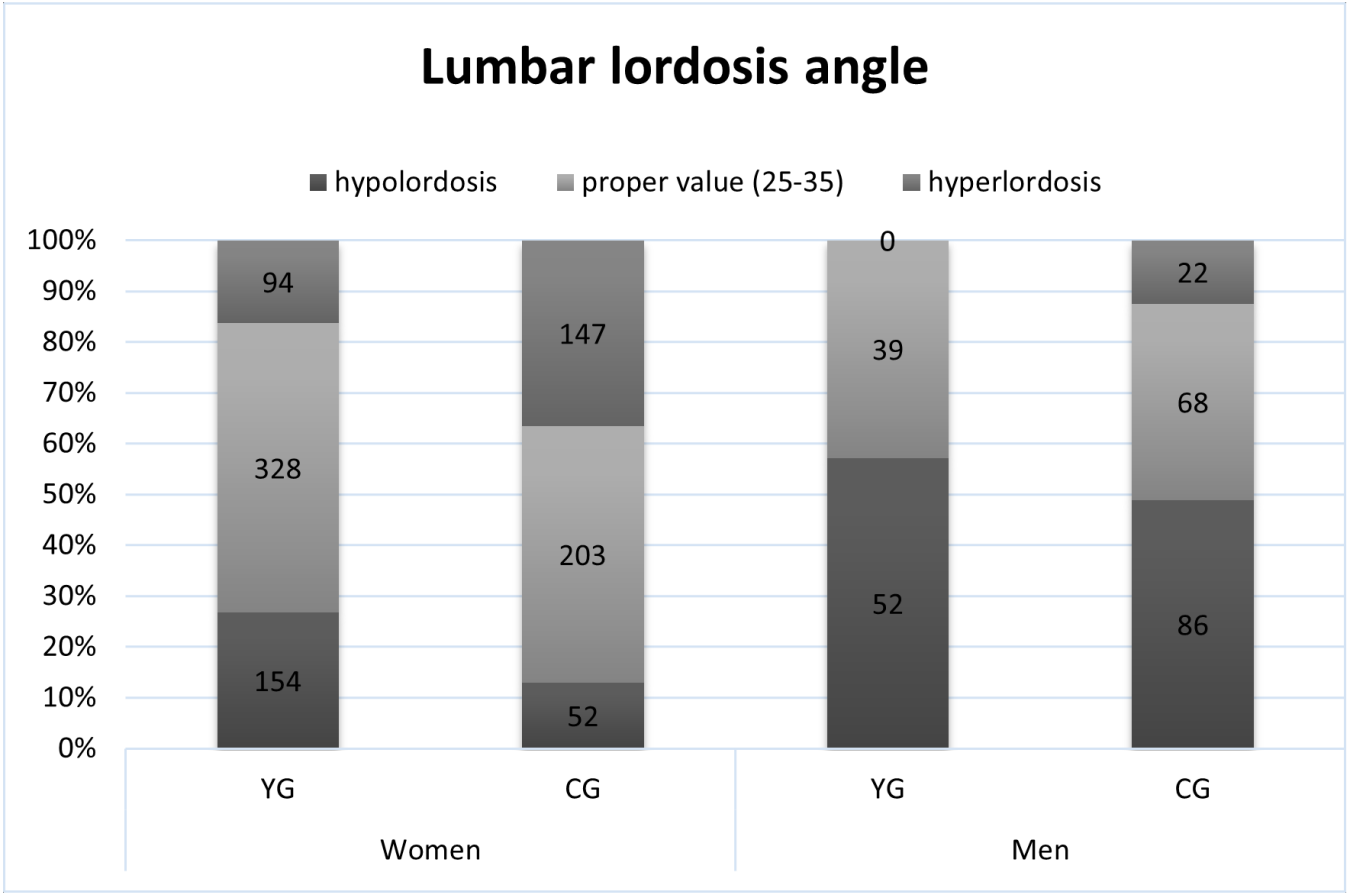
The occurrence of proper lumbar lordosis, hypolordosis and hyperlordosis.

The proper value of the angle of thoracic kyphosis (30 ± 5) was shown in 55% of female and 41% of male yoga practitioners and 44% of female and 48% of male students (Fig. 1). The proper value of the angle of lumbar lordosis (30 ± 5) was shown in 57% of female and 43% of male yoga practitioners and 50% of female and 37% of male students (Fig. 2). Based on Pearson’s chi^2^ test of independence, the frequency of occurrence of proper thoracic kyphosis, hypokyphosis, or hyperkyphosis varied between YG and CG regardless of sex (Chi^2^ = 40.5, *p* < .0001) and did not differ between women and men regardless of group. The occurrence of proper lumbar lordosis, hypolordosis, or hyperlordosis varied between groups (Chi^2^ = 42.3, *p* < .0001) and between men and women (Chi^2^ = 106.5, *p* < .0001).

Based on the analysis of solely the yoga practitioners group, the influence of undertaking other forms of physical activity on the angle of thoracic kyphosis was observed (*p* = .03, effect size = .007, power analysis = .632). The influence of weekly duration of yoga training and training experience was not detected. However, the two-way ANOVA revealed a significant interaction between yoga duration *vs.* yoga experience *vs.* undertaking other forms of PA and the angle of thoracic kyphosis (*p* = .022, effect size = .03; power analysis = .891).

The analysis of spinal curvatures in relation to somatic parameters in YG showed that the angle of thoracic kyphosis was positively correlated with age (0.15, *p* < .0001), body mass (0.16, *p* < .0001), and BMI (0.17, *p* < .0001). The same correlations were found in female yoga practitioners. The angle of lumbar lordosis was negatively correlated with body height (−0.25, *p* < .0001) and body mass (−0.11, *p* = .003). In female yoga practitioners, a positive correlation between lumbar lordosis and body mass (0.11, *p* = .012) as well as BMI (0.19, *p* < .0001) was observed. When analyzing the male practitioners group, no such correlations were observed.

**Table 1:** The mean ± sd, median minimum and maximum values of basic somatic parameters in yoga and control groups.

Parameters			Yoga group (YG)			Control group (CG)			
			Women (*n* = 576)		Men (*n* = 91)		Women (*n* = 402)		Men (*n* = 176)	
Body height [cm]	mean ± sd median min–max		166.2 ± 5.79 165 150–182		178.3 ± 5.7^b^ 177 164–192		166.1 ± 6.31 165.8 151–189		181.2 ± 6.48^b^ 181.5 160–198
Body mass [kg]	mean ± sd median min-max		58.0 ± 6.66 58 41–81		73.5 ± 8.85 71 56–97		58.9 ± 6.8 58.3 38–84.5		73.8 ± 10.15 73.8 49–103
BMI [kg/m^2^]	mean ± sd median min-max		21.00 ± 2.13^a^ 20.7 16–28.2		23.09 ± 2.2 22.96 18.1–29.3		21.31 ± 1.87^a^ 21.31 16.7–25.2		22.47 ± 2.76 22.38 14.7–31.5

**Notes.**

BMI body mass index

a Statistically significant between groups at *p* < .02.

b Statistically significant between groups at *p* < .01.

**Table 2:** The mean ±sd, median, minimum and maximum values of the thoracic kyphosis angle (ThKA) and lumbar lordosis angle (LLA) in yoga and control groups.

Parameters		Yoga group (YG)		Control group (CG)	
		Women (*n* = 576)	Men (*n* = 91)	Women (*n* = 402)	Men (*n* = 176)
ThKA [°]	mean ± sd median min-max	31.4 ± 7.01 30 4–54	33.3 ± 7.83 33 12–50	34.7 ± 7.8 34 18–58	35.2 ± 8.31 33 10–63
LLA [°]	mean ± sd median min-max	28.9 ± 7.29 30 0–58	22.5 ± 6.54 23 0–35	32.8 ± 7.35 32 10–60	26.1 ± 8.1 26 4–50

**Table 3:** The result of post hoc analysis for thoracic kyphosis angle.

ThKA	CG women	CG men	YG women
YG women	***p* < .0001**		
YG men		*p* = .29	*p* = .35
CG women		*p* = .91	

**Table 4:** The result of post hoc analysis for lumbar lordosis angle.

LLA	CG women	CG men	YG women
YG women	***p* < .0001**		
YG men		***p* = .004**	***p* < .0001**
CG women		***p* < .0001**	

Linear multiple regression analysis with the angle of thoracic kyphosis as a dependent variable showed a relationship with age (*p* = .0010, *b* = 0.10), body mass (*p* = .0002, *b* = 0.12), and undertaking other forms of PA (*p* = .0044, b = −1.95), (R = .230707; R^2 ^ = .053226; F(3.647) = 12.12;*p* < .00001; standard error of estimation: 6.968).

Linear multiple regression analysis with the angle of lumbar lordosis as a dependent variable showed a relationship with body mass (*p* < .00001, b = −0.38) and BMI (*p* < .00001, *b* = 1.37), (*R* = .27833; R^2 ^ = .077468; F(2.648) = 27.207; *p* < .00001; standard error of estimation: 7.117).

## Discussion

The angles of thoracic kyphosis and lumbar lordosis determine the spinal alignment in the sagittal plane. The first objective of this study was to compare thoracic kyphosis and lumbar lordosis while taking into account group (yoga practitioners and non-practicing participants) and sex.

The study revealed the influence of group and sex on spinal curvatures but no interaction between group *vs.* sex.

Women presented more pronounced lumbar lordosis than men, but thoracic kyphosis did not differ between men and women. These findings were similar to previous studies. Lang-Tapia et al. in their study including 297 women and 362 men ages 36.6 and 39.8 years old, respectively, observed that men had a smaller lumbar and larger thoracic kyphosis than women4. The same results were obtained Poussa et al. in adults ages 22 years old (Poussa et al., 2005).

Yoga practitioners had a generally less pronounced thoracic kyphosis and lumbar lordosis and were more often characterized by normal or smaller thoracic kyphosis and lumbar lordosis than the students from the control group. This suggests that yoga exercises can affect the shape of the anterior-posterior curves of the spine. Hatha yoga attempts to focus on controlled movements and proper body alignment. Particular attention is given to the elongation of the spine along with the activation of the abdominal muscles accompanied by a reduction in pelvis anteversion and flattening of the spinal curvatures. One of the important aspects of yoga training is postural re-education, *i.e.,* the development and maintenance of correct posture, which is hindered by a number of improper postural habits (Grabara & Szopa, 2011a). What is more, in hatha yoga training, backward bends (straightening exercises) are often performed, which may reduce thoracic kyphosis. Backward bends aim at stretching the muscles of the chest and strengthening the back muscles or the abdominal muscles. Increasing back extensor strength helps decrease thoracic kyphosis (Itoi & Sinaki, 1994), whereas reduced back extensor muscle strength and the weakness of the abdominal muscle and shoulder girdle may be reasons for hyperkyphotic posture (Greendale et al., 2009; Bansal, Katzman & Giangregorio, 2014). On the other hand, focusing on reducing the pelvis anteversion and strengthening the abdominal muscles could lead to a reduction in lumbar lordosis.

Grabara and Szopa (2011) revealed the decrease of thoracic kyphosis and lumbar lordosis in male and female students at the mean age of 19.8 years old who participated in 90-minute hatha yoga classes once per week. The authors also noted that while only 40–45% of the observed participants were characterized by proper values for thoracic kyphosis and lumbar lordosis in the 1 st measurement, after yoga classes the proper values of these angles increased to 56–62%. These results indicated the beneficial effect of yoga exercises on spinal curvatures in young adults (Grabara & Szopa, 2011a). Greendale et al. (2009) in their randomized controlled trial involving 118 women and men with hyperkyphosis aged >60 years observed statistically significant improvements in two hyperkyphosis outcomes after a 6-month yoga intervention. Compared to the control group, participants who were randomized to the yoga group experienced a 4.4% greater improvement in their flexicurve kyphosis angle and a 5% greater improvement in their kyphosis index (Greendale et al., 2009). The study evaluating the effects of 8-month yoga training on thoracic kyphosis and lumbar lordosis in 25 men and women over 55 years old indicated that yoga training leads to an improvement in habitual posture in cases of excessive spinal curvatures. The author observed a decrease in thoracic kyphosis and lumbar lordosis in women and in the joint group (men and women together) (Grabara, 2014b). Bansal et al. (2014) pointed out that targeted spinal extension muscle exercises and yoga may reduce kyphosis among older adults with hyperkyphosis (Bansal, Katzman & Giangregorio, 2014).

The second objective was to determine the possible dependencies between sagittal spinal curvatures and somatic parameters, time spent on yoga exercise, and undertaking other physical activities in yoga practitioners.

The present study revealed the influence of undertaking other forms of physical activity on the angle of thoracic kyphosis in YG. The effect of the weekly duration of yoga training and yoga training experience on the angles of thoracic kyphosis or lumbar lordosis was not found. On the other hand, the influence of the interaction of three factors, *i.e.,* yoga duration, yoga experience, and undertaking other forms of PA, on the angle of thoracic kyphosis was observed. This suggests that people who also participated in other forms of physical activity, spent more time on yoga exercise, and had more years of yoga experience, could be characterized by a lower thoracic kyphosis than who did not.

The results showed a relationship between spinal curvatures and age and somatic parameters in yoga practitioners. It was found that the angle of thoracic kyphosis was positively correlated with age, body mass, and BMI in women and in the entire yoga group. The angle of lumbar lordosis was negatively correlated with body height and body mass in the entire group of yoga practitioners; however, in female yoga practitioners lumbar lordosis was positively correlated with body mass and BMI.

The relationship of spinal curvatures and somatic parameters in adults has been observed in previous studies (Lang-Tapia et al., 2011; Zwierzchowska, Tuz & Grabara, 2020). Lang-Tapia et al. (2011) noted that weight status (non-overweight, overweight, or obese) was associated with thoracic kyphosis and lumbar lordosis. Overweight and obese groups showed smaller lumbar lordosis and larger thoracic kyphosis values than non-overweight. Zwierzchowska, Tuz & Grabara (2020) observed the relationship of lumbar lordosis and body height in male young adults and the relationship of lumbar lordosis and body weight in female young adults. These findings are similar to the present study. However, it should be noted that the cited studies also included obese participants, whereas none of the observed male or female yoga participants in the current study were obese.

Previous studies also indicated the relationship of thoracic kyphosis and age (Lang-Tapia et al., 2011; Milne & Lauder, 1974; Fon, Pitt & Thies, 1980; Ostrowska, Rozek-Mroz & Giemza, 2003). Kyphosis was significantly correlated with age and spinal length. The mean value of thoracic kyphosis increased with age after the age of 50 in women and 55 in men. The authors also observed that the mean values of lumbar lordosis showed a steep fall in men and women over the age range of 55–64 (Milne & Lauder, 1974), however that result was not in line with the present study. Fon et al. noted that the angle of kyphosis increased with age and the rate of increase was higher in women than in men, and this appeared to be more obvious after age 4035. Lang-Tapia et al. observed the association of age with lumbar lordosis and thoracic kyphosis. Older groups (30–39, 40–49, and ≥50 years) presented smaller lumbar lordosis and larger thoracic kyphosis values compared with a younger group (20–29 years) (Lang-Tapia et al., 2011). In spite of this, the present study showed that male and female yoga practitioners, who were older than the control participants, presented a better posture with a smaller thoracic kyphosis. With regard to this result, hatha yoga was found to be an efficient training method for shaping proper posture in adults.

### Limitations of the study

The main limitation of this study is the unequal number of surveyed male and female yoga practitioners. However, this reflects the proportion of women to men in postgraduate studies in “relaxation and yoga” and the yoga instructor’s course. Another limitation is shown by the low value of R^2^ for the regression models. A further limitation is presented by the notable differences in the age of the yoga and control groups. However, the control group ages 18–30 years old was selected intentionally, as the group of persons in the posture stabilization period.

## Conclusions

Male and female yoga practitioners had a less-pronounced thoracic kyphosis and lumbar lordosis and were more often characterized by normal or smaller thoracic kyphosis and lumbar lordosis than students from the control group, who were in a period of posture stabilization.

The study revealed that in yoga practitioners, the angle of thoracic kyphosis was positively correlated with age, body mass, and BMI, and negatively correlated with undertaking other forms of PA. The angle of lumbar lordosis was negatively correlated with body height and body mass.

These results suggest that yoga exercise can affect the shape of the anterior-posterior curves of the spine and may be an efficient training method for shaping proper posture in adults.

## Supplemental Information

10.7717/peerj.12185/supp-1 Supplemental Information 1 Raw data

## Additional Information and Declarations

### Data Availability

The following information was supplied regarding data availability: Raw data is available as a Supplemental File.
